# Multicolor Fluorescent Intravital Live Microscopy (FILM) for Surgical Tumor Resection in a Mouse Xenograft Model

**DOI:** 10.1371/journal.pone.0008053

**Published:** 2009-11-30

**Authors:** Greg M. Thurber, Jose L. Figueiredo, Ralph Weissleder

**Affiliations:** Center for Systems Biology, Massachusetts General Hospital, Harvard Medical School, Boston, Massachusetts, United States of America; University of Milano-Bicocca, Italy

## Abstract

**Background:**

Complete surgical resection of neoplasia remains one of the most efficient tumor therapies. However, malignant cell clusters are often left behind during surgery due to the inability to visualize and differentiate them against host tissue. Here we establish the feasibility of multicolor fluorescent intravital live microscopy (FILM) where multiple cellular and/or unique tissue compartments are stained simultaneously and imaged in real time.

**Methodology/Principal Findings:**

Theoretical simulations of imaging probe localization were carried out for three agents with specificity for cancer cells, stromal host response, or vascular perfusion. This transport analysis gave insight into the probe pharmacokinetics and tissue distribution, facilitating the experimental design and allowing predictions to be made about the localization of the probes in other animal models and in the clinic. The imaging probes were administered systemically at optimal time points based on the simulations, and the multicolor FILM images obtained *in vivo* were then compared to conventional pathological sections. Our data show the feasibility of real time *in vivo* pathology at cellular resolution and molecular specificity with excellent agreement between intravital and traditional *in vitro* immunohistochemistry.

**Conclusions/Significance:**

Multicolor FILM is an accurate method for identifying malignant tissue and cells *in vivo*. The imaging probes distributed in a manner similar to predictions based on transport principles, and these models can be used to design future probes and experiments. FILM can provide critical real time feedback and should be a useful tool for more effective and complete cancer resection.

## Introduction

Diagnostic decisions today are primarily based on molecular markers, and there is continuous research and development of new biomarkers that diagnose diseases more specifically, at an earlier stage, and more rapidly[1]. In the case of cancer, complete surgical resection of neoplasia remains one of the most efficient therapies while postsurgical minimal residual disease has a negative effect on long-term outcome[2]. In parallel, significant advances in microscopy[3] are being applied to continuously improve resolution at the subcellular level. One of the most exciting applications of these advancements is to apply cellular resolution imaging to *in vivo* settings to better understand biology in its correct context (e.g. “intravital microscopy”[4], [5]) or for clinical applications (e.g. “intraoperative imaging”[6]–[8]).

**Figure 1:**
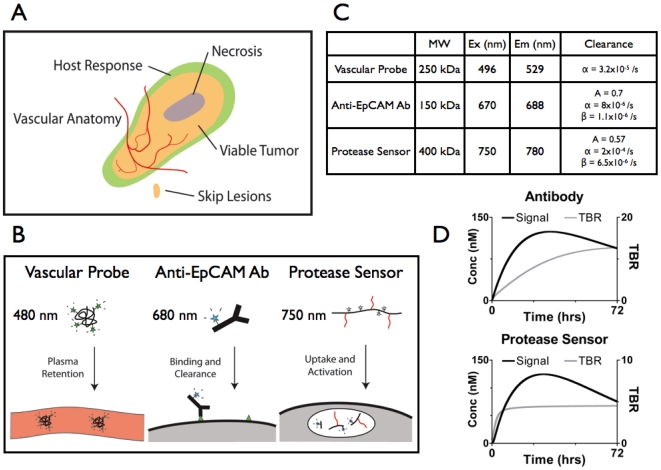
Characterization of Imaging Agents. A) Tumor schematic. B) Mechanisms of probe localization. Numbers indicate wavelength of fluorescent channel. C) Imaging agent properties. MW = molecular weight, Ex = excitation maximum, Em = emission maximum. α and β are plasma clearance rate constants, with A being the fraction of the α phase. D) Simulated time course of antibody and protease sensor localization. Tumor to background ratios were estimated based on a characteristic normal tissue compartment. Different scales are used on the TBR axis.

Genetic markers and manipulation have deepened our understanding of aberrant cancer signaling, growth, and proliferation[9]. Reporter genes involving various fluorescent proteins have allowed the direct visualization of many cellular and subcellular processes, and have opened the door to tracking individual cells within tumors[10]. Such genetic markers have been extremely powerful for experimental research. Unfortunately, they are not easily translated into the clinic, necessitating the need for exogenous reporter probes. While there has been intense interest in universal, highly specific tumor-seeking probes[10]–[13], to date such probes for macroscopic imaging have largely remained elusive. Therefore, FILM for surgical resection will likely require microscopic imaging using a combination of different probes to delineate both tumor and host tissue compartments in the heterogeneous microenvironments found in lesions[14] (figure 1A). While there are several setups being explored for intraoperative imaging of tumor resection (e.g. laparoscopic setup, hand held scanners), all of them involve the ability of the surgeon to simultaneously or quickly image the surgical field using fluorescent markers to identify healthy structures and/or malignant tissue for resection. The molecular markers increase the operator's ability to visualize and remove the tumors while sparing healthy tissue.

Several important questions must be addressed before application of FILM to clinical settings. For example, what is the best route of administration (e.g. intravenous, topical (“tumor paint”), etc.)? What are the kinetics of uptake, distribution, activation, and clearance, and how will these translate to the clinic? What factors dominate the background, and is the signal to background ratio sufficient? Can we obtain cellular resolution in the clinic, and do the *in vivo* results correlate with the current gold standards of diagnosis? These questions must be answered before successful use of FILM in the operating room.

To aid in surgical resection, imaging should be carried out intra-operatively in real time to provide direct feedback to the operator. The surgeon ideally would have a wide field of view to quickly “survey” the tissue, but should also be able to view suspicious regions under higher magnification down to cellular resolution. The imaging probes should target all malignant disease with a sufficient signal to background ratio so that there is little ambiguity between malignant and normal tissue. Ideally, these agents would also identify locoregional metastases for resection. Finally, the agents should be easy to administer so they are readily incorporated into surgical resection procedures.

Topically applied imaging agents have the advantage of using significantly less probe and avoiding blood flow and extravasation limitations (but not diffusion limitations) to transport. However, diffusion of these molecules from the tissue surface to underlying cells is extremely slow[15]. A macromolecule may take a day to diffuse one millimeter from the surface[16]; therefore only exposed superficial cells would be labeled, and lesions deeper in the tissue would be missed. In contradistinction, systemic targeting is able to reach these buried lesions, and timing is less of a concern. We therefore decided to focus on imaging probes administered by intravenous injection well in advance of a planned surgery where the agents use the tumor and peritumor vasculature to transport imaging probe throughout the tissue.

The imaging probes in this study were chosen to highlight three main compartments in the tumor (figure 1B): cancer cells via an epithelial antigen (CD326, EpCAM commonly used to detect circulating cancer cells and overexpressed on a wide variety of epithelial cancers[17]), functional supporting microvasculature (a probe with prolonged distribution in the plasma volume), and stromal tissue (easily targeted innate immune cells, such as the CD11b pool). The antibody localization is based on binding and retention in the tumor tissue, while the protease sensor contains quenched fluorophores. Upon cleavage of the backbone, these fluorophores are de-quenched, yielding a fluorescent signal. These representative imaging agents and other similar molecules are being studied for clinical translation[8], [18]–[22].

This study was designed to address several questions about the feasibility of multicolor FILM using both an experimental and theoretical approach. We argued that the use of multiple markers will provide more complete information to the surgeon, not only conveying the location of neoplastic cells and necrosis, but also the infiltration or encapsulation status of the border regions.

## Results

### Theoretical Analysis

To gain insight into the uptake and distribution of these imaging agents and help interpret the data, a computational model based on the mechanistic steps in transport was developed for each probe. A more thorough understanding of imaging probe localization and limitations provides guidance for how they may be used, and when they may fail, in the clinic. This analysis requires a systems level understanding of the pharmacokinetics, from the whole body plasma clearance (figure 1C) to the molecular and cellular kinetics. Both the antibody and protease sensor must flow to the tumor in the blood, extravasate into the interstitium, diffuse to their site of action, and then bind (in the case of antibodies) or be activated (taken up by pinocytosis and cleaved in the case of the protease sensor). The kinetic rate for each of these steps for this particular antibody and protease sensor were analyzed in the model (supplemental information S1). Using this model for the tumor and normal tissue uptake, the concentration and tumor to background ratios of the imaging agents were simulated over time (figure 1D). Based on these simulations, the imaging time points of 24 hours for the protease sensor (primarily a marker for the CD11b(+) pool) and 72 hours for the tumor antibody were chosen based on total signal. While the maximum uptake was estimated after ∼1 day for the antibody, there was uncertainty in the internalization rate from the literature, with slower rates yielding peak uptake at later times.

**Figure 2:**
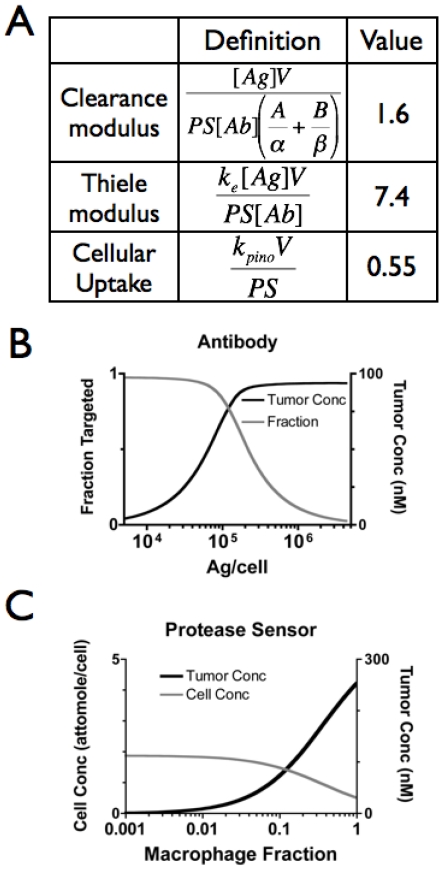
Theoretical Localization. A) Dimensionless groups that describe the fundamental limits to antibody uptake (clearance and Thiele modulus) and protease sensor localization (cellular uptake number). A clearance modulus or Thiele modulus greater than one indicates the antibody is cleared from the plasma too rapidly or internalized by the cells too quickly to saturate the tumor. A cellular uptake number less than one means the protease sensor will localize based on the local pinocytosis rate instead of vascular transport. B) Antibody simulation. At subsaturating doses, antibody uptake is not sensitive to target/antigen density. C) Protease sensor simulation. In contrast to the antibody, the concentration is dependent on the number of infiltrating macrophages, not the extent of vascularization.

Based on theoretical considerations, a 30 µg/mouse dose for this antibody and model is subsaturating even in these small, well vascularized tumors. The plasma concentration (AUC) was not quite sufficient to target all EpCAM, since the clearance modulus was greater than one (figure 2A, table 1). A clearance modulus less than one would indicate the antibody has sufficient time in the plasma to extravasate and target all the tumor cells. Even with slower clearance, the 15-hr half-life of internalization sequesters enough antibody to prevent targeting of all antigens. A Thiele modulus greater than unity signifies that the antibody turnover (internalization and degradation) in the tissue is faster than the rate at which antibody enters the tumor. Both of these values must be less than one for saturation to occur[23].

**Table 1:** Model Parameters.

Probe	Parameter	Symbol	Typical Value
Antibody	Antigen concentration	[Ag]	1.7 µM
	Antibody concentration	[Ab]	100 nM
	Vascular permeability	P	3×10^−9^ m/s
	Blood vessel surface area to volume ratio	S/V	100/cm
	Biexponential clearance rate constants	A	0.7
		B	0.3
		α	8×10^−6^/s
		β	1.1×10^−6^/s
	Antigen internalization rate constant	k_e_	1.3×10^−5^/s
Protease Sensor	Fluid pinocytosis rate	k_pino_	1.1×10^−5^/s
	Vascular permeability	P	10^−9^ m/s
	Blood vessel surface area to volume ratio	S/V	200/cm

With subsaturating doses, the antibody concentration in the tumor has low sensitivity to the number of binding sites. To show this, the antibody concentration in the tumors was simulated as a function of the number of targets per cell. For these molecules, blood flow, diffusion, and binding all occur much faster than extravasation, which is the rate-limiting step in uptake[16]. Using a combination of parameters measured *in vitro* and estimated from the literature (supplemental information S1), the antibody concentration in the tumors was simulated[23]. With a large number of targets per cell, as with many cancer antigens (e.g. EGFR, HER2/neu, CEA), the number of free binding sites exceeds the number of antibodies entering the tissue. At this subsaturating dose, the tumor concentration depends on the extent (surface area) of functional blood vessels in the tissue and is insensitive to the number of binding sites. In contrast, with a low number of antigens per cell the tumor becomes saturated, and the additional antibody that enters the tumor interstitium has no free binding sites. It therefore clears from the tissue, and the concentration becomes a function of the antigen density (figure 2B). The transition between these two regimes is dependent on multiple factors present in the clearance and Thiele modulus.

The kinetics of a model protease sensor[24] localization were also examined. These agents are taken up through pinocytosis by the cell and cleaved, de-quenching their fluorescence. The probe may also be activated by secreted proteases, but due to the high endocytosis rate of activated macrophages, the model assumed that intracellular probe dominated the signal. Based on theoretical estimates (supplemental information S1), the cleavage kinetics of the probe are faster than cellular uptake by activated macrophages, so the enzyme kinetics were ignored. With antibodies, the antigen binding rate is much faster than diffusion, immobilizing the antibody in a perivascular distribution. For the protease sensor, the diffusion rate is faster than cellular uptake. The probe therefore distributes more evenly in the tissue. In fact, the overall rate of pinocytosis[25], which is a function of how many cells are ingesting fluid in the tissue, is also slower than blood flow and extravasation, as indicated by the cellular uptake modulus less than one (figure 2A). This ratio of cellular uptake to vascular transport indicates that pinocytosis is slower than delivery. The uptake of the protease sensor is therefore a function of the fraction of macrophages (which have a high phagocytosis rate) in the tissue (figure 2C), with the uptake per cell being relatively constant, as seen experimentally in other systems[26]. Only when macrophages form 10 to 100% of the tissue does the uptake per cell start to slow down due to limitations in extravasation of the probe.

The vascular probe used was a macromolecule imaged at early time points following injection before the plasma concentration drops due to redistribution in the tissue. Similar to the other macromolecules, the blood flow rate in most vessels is faster than extravasation for this agent, allowing the probe to fill the full length of the vessel. Interstitial diffusion is also faster than extravasation, so any probe that does leak out diffuses away from the vessel, giving a clean outline.

### Cellular Localization

**Figure 3:**
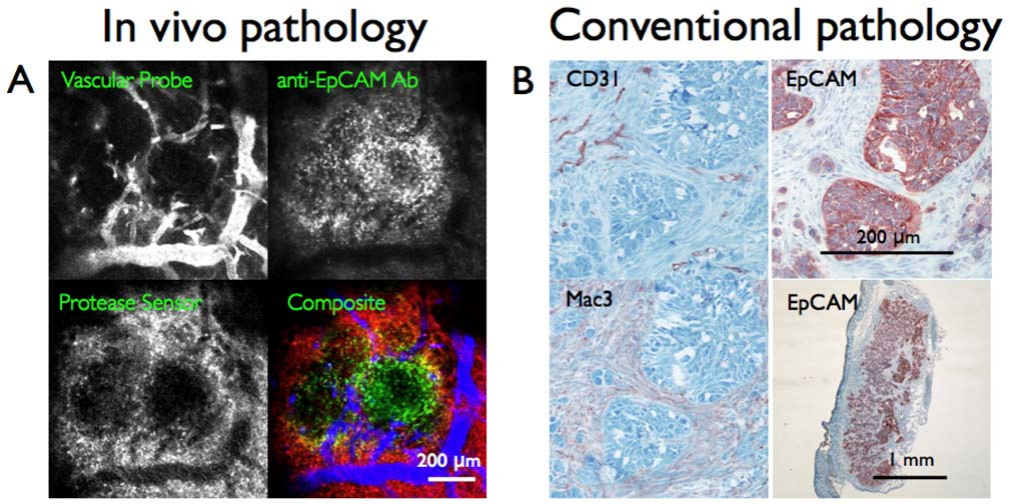
Cellular Localization. A) Multichannel *in vivo* image of HT-29 tumor. The vascular probe outlines the blood vessels, anti-EpCAM antibody labels tumor cells with some macrophage binding, and the protease sensor localizes to tumor macrophages. B) Histology of the samples with CD31 labeling blood vessels, anti-EpCAM labeling tumor cells, and Mac3 labeling tumor macrophages. The low magnification EpCAM image shows the heterogeneous distribution of cells in the tumor.

After simulating the results, the technique was tested in a subcutaneous colon cancer model. Using a 10X objective for multicolor FILM, the vascular probe yielded well-delineated blood vessels (figure 3A). The antibody localized primarily in clusters of tumor cells, but it also resulted in a significant signal in the surrounding stromal tissue (presumably host cells). The anti-human EpCAM antibody is a mouse IgG_2_ class molecule, and the Fc region, which helps increase plasma half-life through FcRn interactions, also binds to several types of immune cells[27] when injected intravenously (an important contradistinction to frozen sections where this interaction can be blocked). Flow cytometry of digested tumors indicated the stromal cells were primarily tumor-associated macrophages, and the antibody bound to these cells *ex vivo* (data not shown). However, the majority of retention occurred due to EpCAM binding, since non-specific antibody controls had drastically lower uptake. The protease sensor was localized primarily in a thin palisade of tumor stromal cells. Further analysis revealed that the probe accumulated preferentially in macrophages due to the high cellular uptake rates and abundant expression of cathepsin B[28]. Some cancer cell lines have been shown to take up protease sensors[29], and this uptake has been correlated with tumor aggressiveness[30]. This is not surprising since cell growth and proliferation signaling networks are connected to cellular trafficking[31]. Based on these results, the moderately differentiated HT-29 cell line appeared to have a lower cellular uptake compared to other experimental models.

The color FILM imaging results were compared with immunohistochemical staining, the gold standard for tissue pathology. Anti-CD31 staining showed vessels that were exclusively located in the stroma, which stained primarily with a Mac3 antibody. The stromal tissue surrounded clusters of cancer cells, which stained with an anti-EpCAM antibody (figure 3B).

### Tumoral Heterogeneity

**Figure 4:**
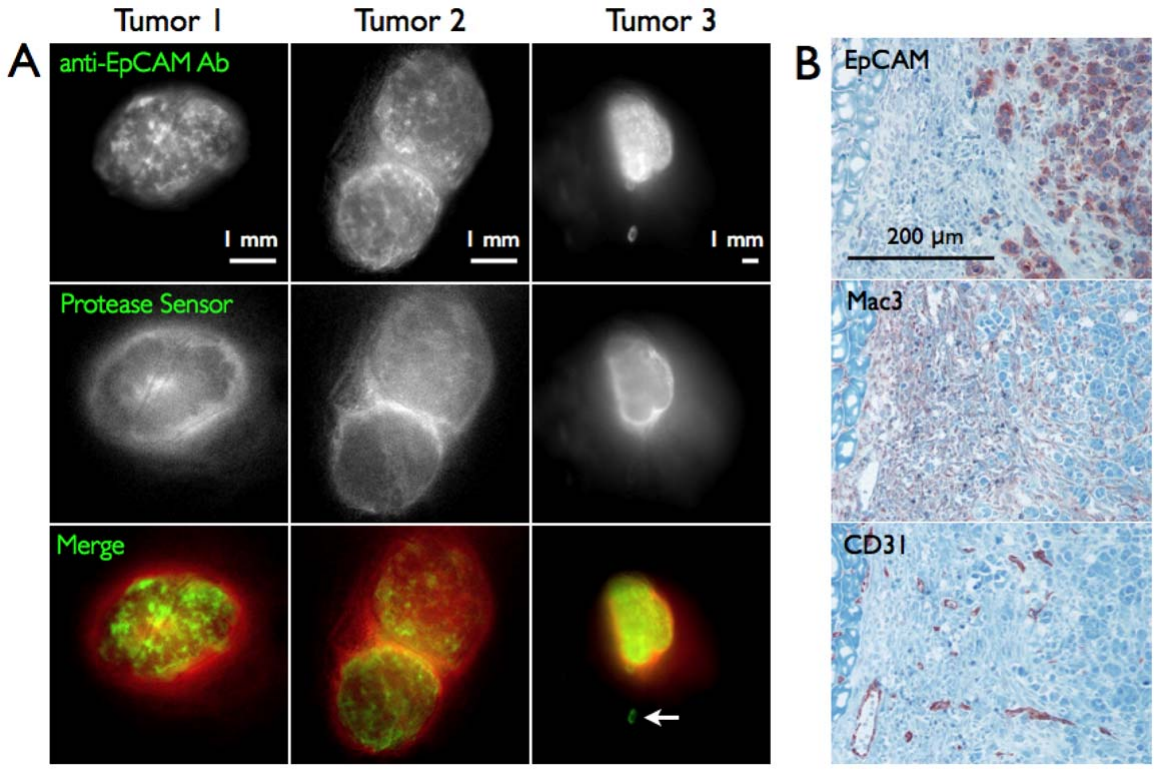
Tumor Heterogeneity. A) Low magnification epifluorescence image of tumors. A skip lesion is highlighted with an arrow in tumor 3. B) Histology of the tumor border showing a well vascularized ∼200 µm thick tumor capsule between the underlying muscle (left) and tumor cells (right).

To address the issue of pan-tumoral distribution, color FILM images of entire tumors were acquired with a 0.56X objective (figure 4A). The different tumors (even within the same animal when implanted into different sites) were highly heterogeneous due to the stochastic nature of tumor cell growth, vascularization, necrosis, stromal cell infiltration, etc. The antibody localized in a heterogeneous pattern near the surface of the tumor, but with no optical slicing, much of the signal was scattered from cells deeper in the tissue. The protease sensor gave a moderate signal in the middle of the tumor surrounded by an intense ring.

Histology at the surface of the tumor (figure 4B) showed a highly vascularized, well-encapsulated tumor. The capsule stained primarily with Mac3, indicating a high concentration of infiltrating macrophages between the tumor cells and underlying muscle. Based on CD31 staining, the blood vessel surface area to tumor volume was ∼260 cm^2^/cm^3^ in this outer region, whereas it was ∼120 cm^2^/cm^3^ in the middle of the tumor. The actual disparity may be even larger, since CD31 staining does not indicate which vessels are functional. The vascular probe showed mostly functional vessels near the tumor surface, but vascular collapse and poor blood flow may occur near the center. Based on the theoretical simulations, however, the higher protease sensor uptake in the ring-like pattern is due to the high macrophage density in the capsule, not increased blood flow or extravasation in the periphery.

### Specificity

**Figure 5:**
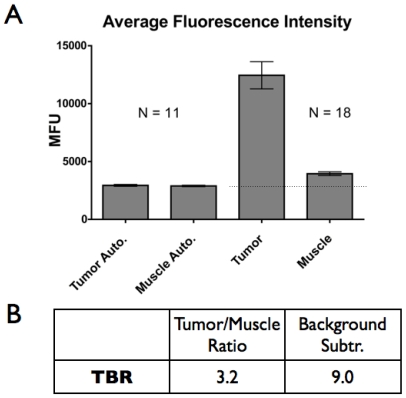
Signal Intensity. A) Semi-quantitative epifluorescence of antibody signal. The average intensity value for the ROI is reported with the standard error. N = number of tumors. B) Tumor to background ratios based on total signal intensity and background subtracted signals.

The signal-to-background ratio for these probes is important in the intraoperative setting in order to distinguish cancerous cells from healthy tissue. Region of interest analysis was done on 4 control mice and 5 experimental mice to compare tumor to background levels. A large field of view is desirable for scanning the surgical field, so a 0.14X objective was used for analysis (6.3 cm by 4.7 cm field of view). The autofluorescence (no injected probe) of the tumors and healthy surrounding tissue in the near infrared was similar (figure 5A). When mice were injected with anti-EpCAM-680, the fluorescence signal increased dramatically for the tumors, while non-specific uptake in healthy surrounding tissue was minimal. The fluorescence ratio between tumor and muscle after probe administration in the mice was 3.2 (figure 5B), so the tumor could clearly be visualized against the background tissue without any correction for autofluorescence. However, by subtracting away the autofluorescence, the ratio between the fluorophore in the tumor versus the muscle was 9 to 1. This is reasonable compared with the predicted ratio of 13 from the theoretical simulations based on fluorophore concentrations. While the signal adjusted for autofluorescence gives the larger contrast and more accurately reflects the specificity of the probe, this adjustment becomes problematic with tissues that have widely varying autofluorescence levels. Ideally, the level of autofluorescence would be known for a given tissue, allowing the baseline signal to be adjusted accordingly.

The relationship between tumor-to-background ratios and magnification is complex and a result of several factors. In general, higher magnification yields better contrast due to increased efficiency in exciting and capturing light with higher numerical aperture lenses and lower partial volume/pixelation effects in small metastases. With cellular resolution, contrast between tumor cells and background increases further due to cellular localization. While the background results from diffuse unbound probe in the case of an antibody, specific binding on tumor cells yields intense punctate labeling. With the optical slicing ability of a laser-scanning microscope, the background of out-of-focus light is also reduced. Tumor-to-background ratios with cellular resolution in the laser scanning system are routinely greater than 10 with no adjustments for autofluorescence, compared to 3.2 with macroscopic imaging.

## Discussion

Fluorescence imaging is expected to become a powerful adjunct to traditional surgical oncologic therapies. To become a reality, several technological advances and proof-of-principle studies are necessary. These include instrumentation and visualization techniques[8], imaging probes for human use[32], and preclinical efficacy testing in different models. Several of these questions were addressed in these experiments through the development of multicolor FILM.

### Clinical Requirements

The successful application of multicolor FILM for surgical resection will require biocompatible and safe probes to clearly identify malignant cells *in vivo* in real time. This is advantageous over frozen sectioning by maintaining the exact spatial location of the unresected cancer, rather than inferring the remainder of cells at the surgical margins. Such cancer cells should ideally be identifiable based on molecular markers of the disease, rather than structural indirect information. Early results with intraoperative imaging have shown successful identification of lesions that were otherwise undetectable[33]. However, localization based on structural abnormalities resulted in several false positives, highlighting the need for more specific imaging agents. While pan-carcinoma markers, such as EpCAM, should target many cancers, pre-operative biopsies could be used to identify appropriate markers, similar to the indication for trastuzumab. Cell surface antigens that are overexpressed at a high level with little to no expression in normal tissues would be ideal targets, but expression levels vary considerably among patients and even between lesions, which is an additional reason why a multichannel approach of host response was used in parallel.

### Delivery

The agents used in this study were delivered systemically to target malignant cells, host tissue infiltration, and the tumor vasculature. While the ease of delivering a topical agent is appealing, penetration of these agents is severely limited. Due to the slow diffusion of molecules in tissue, the maximum penetration depth would be a few hundred microns. An effective topical agent would have to be able to be delivered quickly (i.e. minutes), so that it could be re-applied several times after resection to verify clean margins. On this time scale, high doses of macromolecule are required to overcome the reduction in penetration due to binding. At these doses, probe passively taken up by healthy tissue in the interstitium or non-specifically bound is not able to be cleared quickly, resulting in poor tumor to background ratios (data not shown).

For the systemically delivered agents, transport simulations were used to help design the experiments and make predictions on distribution. These simulations showed that the antibody dose was sub-saturating and therefore limited by delivery (vascular surface area and permeability) rather than the number of binding sites. While the antigen expression level does not have a large impact on antibody signal with subsaturating doses, high antigen expression is still ideal since it will yield a brighter signal when targeting small metastases and single cells, which are more easily saturated. The extravasation limited antibody uptake in tumors is in contrast to the protease sensor, where cellular uptake was slower than vascular delivery. In this case, the signal intensity is proportional to the fraction of macrophages that are actively taking up the sensor.

Using subsaturating doses of antibody, some cancer cells may not be targeted, especially in poorly perfused regions. However, the well vascularized border regions[34] and diffusion inward from the surface of tumors and micrometastases[35] ensure adequate targeting of these critical border areas. In fact, a small skip lesion, which could be missed in frozen sections, was easily visualized with the antibody (figure 4, arrow).

The antibody gave a strong signal to background ratio, even at subsaturating doses. In theory, the tumor to background ratio is not dependent on the dose, since both signals are proportional to the amount of probe injected. For example, doubling the dose will double the tumor signal (prior to saturation) and double the non-specific signal in normal tissue[36]. However, the instrument noise and autofluorescence are constant, so the dose must be large enough to yield a signal significantly higher than this background. Achieving a signal above this autofluorescence is the main limitation to lowering the dose. Antibodies maintain their specificity even at doses 1000-fold lower than used here (e.g. nanograms of radiolabeled antibody/mouse[37]), so methods to reduce autofluorescence or amplify the signal (spectral deconvolution, up-converting fluorophores, click chemistry amplification, etc.) could lead to lower required doses.

### Magnification and Resolution

In the intraoperative setting, the operator must be able to image the entire surgical field to identify tissue that needs to be resected. The tumors were easily visualized with a low magnification objective (0.14X) giving a field of view of 30 cm^2^. However, after resection, the border regions may be scanned at high magnification to achieve cellular resolution. Instrumentation to quickly switch to higher magnification objectives would be ideal to detect any remaining cells at the surgical margins by allowing suspicious areas to be examined more closely without requiring a time consuming scan of the entire surgical field. The larger numerical aperture of these higher magnification lenses is able to capture the light more efficiently from the sample, detecting individual cells.

The laser scanning color FILM images in figure 3 illustrate the distribution of the different probes without the diffuse background from signal deeper in the tumor. While these images were taken at the surface of the tumor, the optical slicing is likely not needed when detecting a few isolated cells, for example at a positive margin, since there will be little signal from deeper in the healthy tissue. In the clinical setting, a diffuse signal could indicate a tumor mass buried under healthy tissue. Detection of buried lesions depends on several factors, such as the size of the tumor mass, efficiency of probe uptake (e.g. vascularization), wavelength of light, and type of tissue. At this point, it is not possible to detect a few cells buried under millimeters or centimeters of tissue, but microscopic imaging is able to detect cells at an exposed resection margin. A number of fiberoptic systems with the above capabilities are currently under development.

### Multicolor Imaging

The systemically delivered agents were able to identify separate compartments within the tumor. Color FILM using a 10X objective showed the antibody was able to target cancer cells based on expression of the epithelial cell lineage marker EpCAM, while the vascular probe and protease sensor provided feedback on the extent of vascularization[38], [39] and stromal cell infiltration[40]. Although the malignant cells are an important target for resection and can help distinguish tumor cells from inflamed tissue, the other imaging agents put the antibody probe in context. Regions devoid of antibody but also lacking vascular probe may indicate poor blood flow and necrosis. This tissue can be resected to remove any remaining viable cells in these hypoxic regions. Depending on the tumor, malignant cells at the border regions could be well encapsulated by stromal tissue or infiltrating into healthy adjacent tissue as indicated by the protease sensor. The latter case may require further inspection under higher magnification to ensure adequate margins are taken. This information would not be provided to the operator using single channel (black and white) FILM.

### Challenges

This study demonstrates the feasibility of color FILM for tumor resection. Separate cancer cell, vascular, and stromal compartments were imaged *in vivo* from the whole tumor to the cellular level, and a signal to background ratio of 9 provided clear contrast between malignant and healthy tissue. Several issues need to be explored further before transitioning into the clinic. First, is the tumor to background ratio sufficient in multiple types of cancer with different antigens since the autofluorescence and expression levels vary? Second, can images be taken at video frame rates, allowing simultaneous imaging and resection? Are there problems when dealing with different organ systems, such as imaging internal body cavities or delivery to different tissues (e.g. crossing the blood brain barrier)? Finally, will these probes be useful for detecting lymph node metastases? These issues are currently being examined with additional experiments.

In conclusion, real time, multicolor FILM is capable of providing critical information on the location of malignant cells, the status of border regions, and extent of local metastasis for surgical oncology. Identification of malignant tissue can be done instantaneously with the exact spatial location of residual cells including skip lesions, advantages over frozen sectioning during surgery. A computational model was developed to describe the localization of these probes, and this model was used to help design the experiments, interpret the results, and make predictions of behavior in other systems. Multicolor FILM has the potential to facilitate complete resection, obtaining negative surgical margins to lower morbidity from local recurrence[2], [41]–[43] and decrease mortality[44].

## Materials and Methods

### In vivo Imaging

All animal experiments were carried out in accordance with guidelines from the Massachusetts General Hospital Subcommittee on Research Animal Care which serves as the institutional animal care and use committee as required by the Public Health service. Mice were maintained on a non-fluorescent diet throughout all experiments. For the tumor mouse model, the moderately differentiated human colon adenocarcinoma line HT-29 (ATCC; Manassas, VA) which expresses 2–3 million EpCAM per cell was used. Approximately 1.5 million cells were injected subcutaneously in nude mice (Cox-7, Massachusetts General Hospital, Boston, MA, USA). When the tumors reached 3–4 mm in size, mice received 30 µg of anti-EpCAM antibody (R&D Systems; Minneapolis, MN) conjugated to VT-680 dye (Visen; Bedford, MA) by tail vein injection three days before imaging. Antibodies contained approximately 2 dye per IgG. One day before imaging, mice received 2 nmol of Prosense-750 (Visen; Bedford, MA) via tail vein, and Angiosense-488 was administered retro-orbitally immediately before imaging.

Mice were anesthetized with intraperitoneal injection of 90 mg/kg ketamine and 10 mg/kg xylazine, and the skin covering the tumor and surrounding muscle was removed. An Olympus IV-100 system (Olympus; Center Valley, PA) was used to image the 488 nm, 680 nm, and 750 nm channels in series using an Olympus 10X/NA 0.4 objective. A metal stabilizer ring was required to minimize respiratory and cardiac motion artifacts. An Olympus OV-110 epifluorescence imager was used for whole tumor imaging. The gain on the camera was set to zero and a 280 ms exposure time was used for semi-quantitative analysis. Longer exposure times sometimes saturated the image, while shorter exposures provided good contrast but did not use the full dynamic range of the camera for quantification. Under the conditions used, the fluorescence intensity was linearly proportional to the dye concentration in controls (data not shown).

### Tumor Characterization and Histology

The number of EpCAM binding sites per cell was measured using flow cytometry with quantitative beads (Bangs Laboratories; Fishers, IN) according to the manufacturer's instructions. The blood vessel surface area per tumor volume was estimated from the CD31 stained slides using the method of Hilmas and Gilette[45].

After imaging, tumors were flash frozen in OCT using isopentane cooled with dry ice. The serial histology slices were stained with an anti-EpCAM antibody (R&D Systems; Minneapolis, MN), Mac3 antibody (BD Pharmingen; Franklin Lakes, NJ), and anti-CD31 antibody (BD Pharmingen; Franklin Lakes, NJ) using standard protocols.

For flow cytometry on tumor cells, a protocol was adapted from one previously described[46]. Briefly, the tumors were excised from the mice, digested in 0.2 mg/mL collagenase IV (Sigma-Aldrich) for 1 hr at 37°C, filtered through a 40 µm nylon mesh, washed, and resuspended in PBS prior to analysis.

## Supporting Information

Supplemental Information S1 (0.87 MB DOC)
