# Supplementary material for: Multicolor Fluorescent Intravital Live Microscopy (FILM) for Surgical Tumor Resection in a Mouse Xenograft Model
Source: PLoS One. 2009 Nov 30;4(11):e8053. doi: 10.1371/journal.pone.0008053 (PMC2779447; doi:10.1371/journal.pone.0008053)
Supplement: Supplemental Information S1 — (0.87 MB DOC) [file pone.0008053.s001.doc]

**Supplemental Information**

This section provides a detailed description of the model development, parameter values, and simulations used for the theoretical uptake predictions. The first section discusses the protease sensor model followed by the second section on antibody targeting.

*Protease Sensor Model*

A model was developed for localization of the protease sensor (PS) based on transport mechanisms. After intravenous injection, the PS must flow through the tumor blood vessels, extravasate across the capillary wall, diffuse to the site of action, be internalized by activating cells, and get cleaved by enzymes. The inactive PS is continually cleared from the plasma and is exchanged with the tissue, while the activated PS eventually clears from the cells. A schematic of these steps is illustrated in figure S1.

Figures S1 – Flow Chart of Protease Sensor Uptake


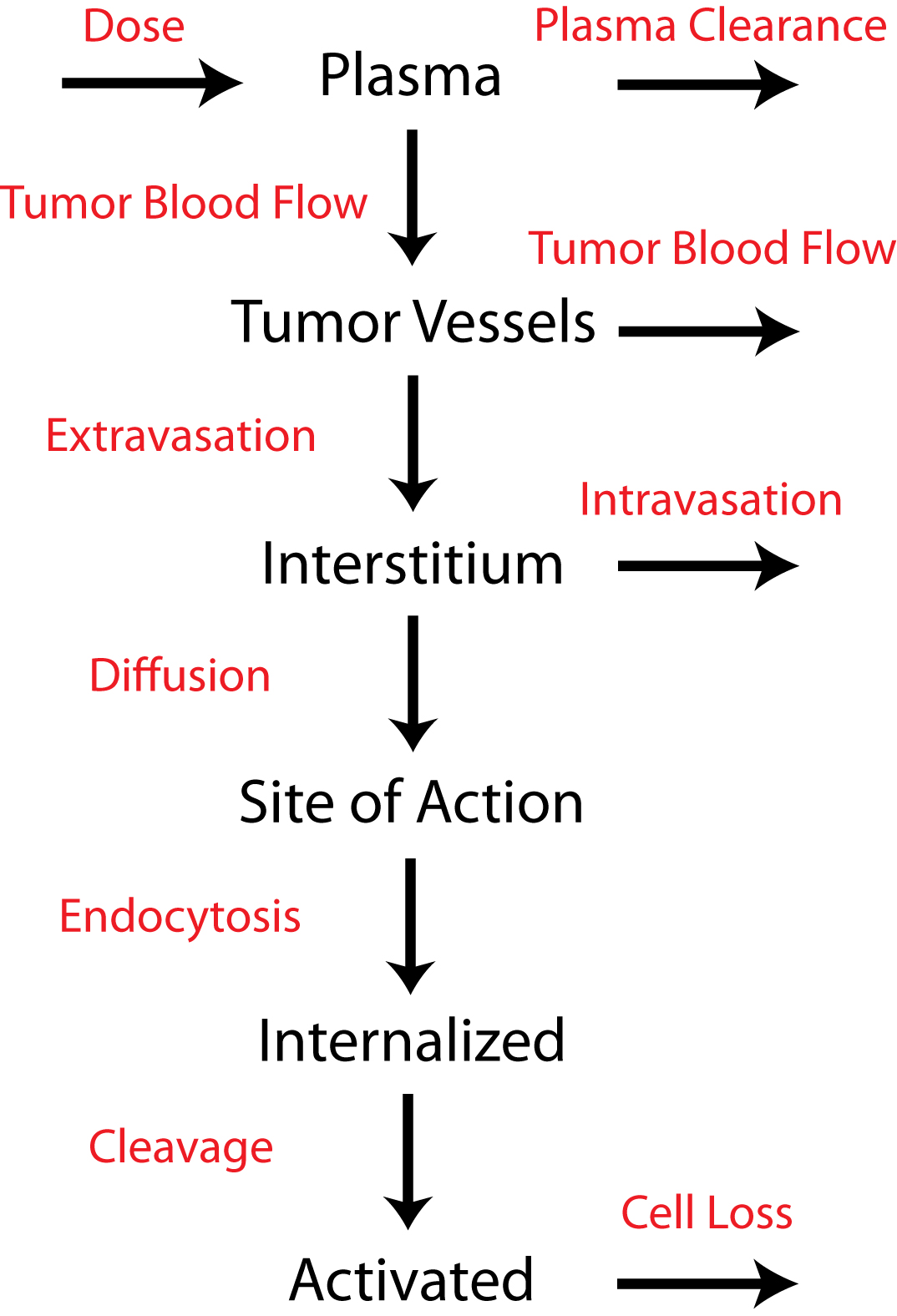


Each of the rates can affect the uptake of PS in the tumor. Using parameters from the literature, the magnitude of each step was estimated. The results are shown in table S1.

Table S1 – Mechanistic Uptake Rates for Protease Sensor

| Step | Characteristic Time | Typical Value | Refs | Note |
| --- | --- | --- | --- | --- |
| Blood Flow |  | ~10 min | [1] | Independent of molecule size |
| Extravasation |  | ~15 hrs | [2,3] | Molecule size dependent |
| Diffusion |  | ~30 min | [4] | Molecule size dependent |
| Endocytosis |  | 5-500 hrs | [5-9] | Dependent on concentration of macrophages in tissue |
| Cleavage |  | ~5 min | [10-12] | Based on enzyme concentration in endosomes and Michaelis-Menten kinetics |

where Q is volume of whole blood per tumor volume per time, Ht is the hematocrit, S/V is the blood vessel surface area per tumor volume, P is the permeability of the vessels, R is the half distance between blood vessels, D is the diffusion coefficient, ε is the void fraction, k_pino_ is the endocytosis rate, K_M_ and k_cat_ are the Micahelis-Menten enzyme kinetic parameters, and [E]_0_ is the vesicle enzyme concentration.

The pinocytosis rate was calculated from the fluid phase endocytosis:

Based on these rates, the PS uptake is not highly dependent (i.e. low sensitivity) on blood flow, diffusion, or enzymatic cleavage rates. This has several implications. First, the blood flow rate is large relative to extravasation. This means that the concentration of PS in functional vessels (i.e. flow rate > 0) is generally not depleted along their length, and the majority of probe flows back out of the tumor in the vessels. Second, the cellular uptake rate is slow relative to diffusion. The probe therefore distributes relatively homogeneously throughout the tissue. In contrast, antibodies bind in seconds, immobilizing them immediately upon extravasation in a perivascular fashion. Finally, the cleavage/activation rates are estimated to be much faster than pinocytosis. Therefore, the concentration is not dependent on enzyme kinetics.

The flow chart can be simplified based on the above scaling. Blood flow and enzyme kinetics can be ignored, whereas extravasation and cellular uptake have similar rates, so both can affect the uptake. The lack of spatial gradients allows the use of a compartmental (lumped parameter) model.

An additional assumption in the model is that extracellular activation is negligible compared to intracellular activation. This assumption is based on the idea that the extracellular fragments may clear faster, and therefore have a lower concentration, than activated probe trapped inside cells. The scaled flow chart is shown in figure S2.

Figure S2 – Scaled Protease Sensor Flow Chart


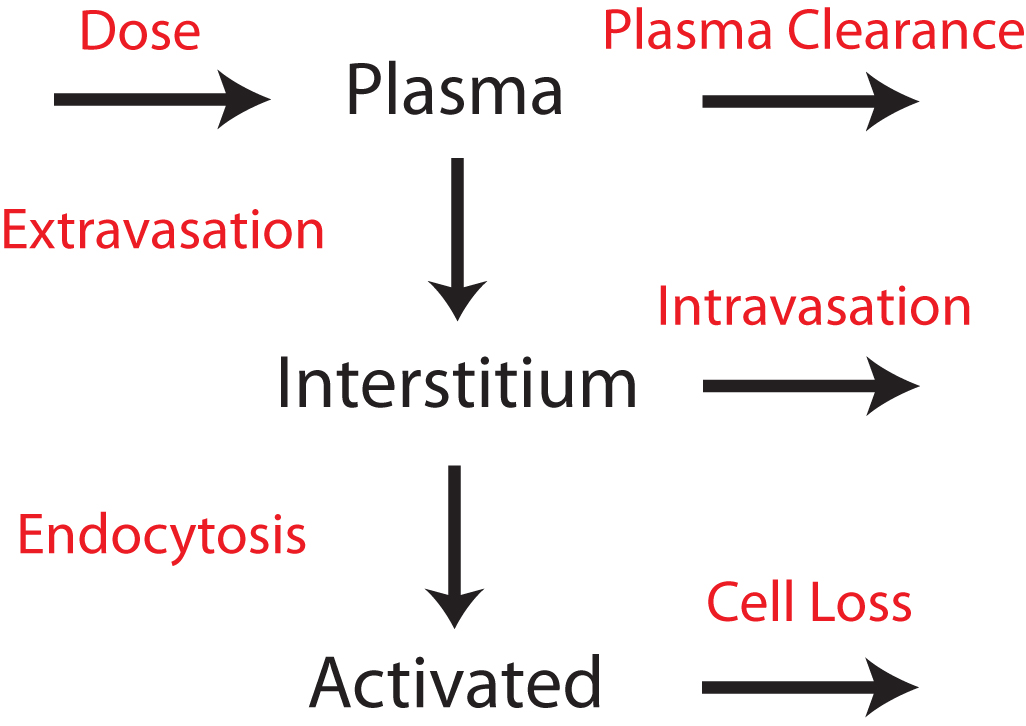


While the structure of this compartmental model is similar to models for FDG uptake, the rates and mechanisms are different for the protease sensor macromolecule compared to the small molecule FDG. In addition, due to the longer clearance half-life and imaging times, the uptake cannot be assumed to be irreversible (i.e. there is loss from the cell), so uptake cannot be linearized over the course of the experiment. The differential equations for the tumor interstitium and internal probe that define the three compartment model (plasma, tumor interstitium, and internal/activated probe concentration) are:

where [I]_i_ and [I]_p_ are the inactivated PS concentrations in the interstitium and plasma, respectively. [A] is the activated (unquenched) PS concentration within the cells. The inactive and activated concentrations are overall concentrations (mol/tumor volume).

The transition between a transport limited regime and a cellular uptake limited regime can be seen by non-dimensionalizing the interstitial PS concentration:

where: and

The dimensionless group $f_{\text{pino}}$ relates the cellular uptake rate to vascular exchange. For values much greater than one, cellular uptake is faster than exchange, and the signal intensity will be limited by transport to the tissue. When the value is much less than one, the rate of pinocytosis will determine the amount of activated PS in the tissue.

Assuming tumor uptake does not affect the plasma concentration (i.e. no TMDD[13]) and internalization is irreversible, the integrated equations are:

where:

In order to estimate the tumor to background ratio (TBR), a simple one-compartment model of ‘normal tissue’ was used. While uptake and clearance varies between organs, as seen in physiologically based pharmacokinetic models[14], the intent here was to estimate the order of magnitude of the TBR to guide the experimental design of the imaging time points.

It is assumed that cells in normal tissue have a low level of pinocytosis, which was estimated based on fibroblast fluid phase uptake. The concentration of inactive and active protease sensor in normal tissue is given by the following equations:

where κ and λ are the capillary extravasation and lymphatic clearance rates, respectively. Similar to the tumor derivation, the equations can be integrated to yield the concentration with time:

where

and

where

With these formulas, the concentration of active and inactive reagent is known for both the tumor and normal tissue. The TBR accounting for PS in the blood, interstitium, and activated fraction is:

where φ_blood,tumor_ and φ_blood,normal_ are the volume fractions of blood in the tumor and normal tissue, respectively. While blood volumes vary, 0.2 and 0.1 are reasonable values for normal and tumor tissue.

The parameters used for the PS model are given in table S2.

Table S2 – Parameters for Protease Sensor Simulations

| Parameter | Symbol | Value | Notes |
| --- | --- | --- | --- |
| Antibody Dose | [I]_p,0_ | 1 μM | Dependent on amount injected |
| Cell density | ρ_cell_ | 5x10^8^ cells/mL | This is total cell density, not macrophage density |
| Macrophage fraction |  | 0.2 | Varied in Figure 4 |
| Pinocytosis rate  (macrophage) | r_pinocytosis_ | 4 μL/hr/10^7^ cells | Average from several macrophage literature values [15] |
| Pinocytosis rate  (fibroblast) | r_pino,norm_ | 0.3 μL/hr/10^7^ cells | Average value for fibroblast[16,17] (estimate for normal tissue) |
| Pinocytosis rate constant | k_pino_ | 1.1x10^-5^ /s | Dependent on macrophage fraction. Units are volume of interstitium per volume of tumor per time |
| Permeability | P | 10^-9^m/s | Dependent on molecular weight |
| Blood vessel surface area | S/V | 200/cm | Varies depending on tissue. Stromal tissue may be very high (> 200/cm), where large necrotic tumors are low (< 20/cm) |
| Diffusion coefficient | D | 10 μm^2^/s |  |
| Void fraction | ε | 0.2 | Varies depending on extracellular matrix and cellular density |
| Blood Flow | Q | 3x10^-3^/s | Flow rate of whole blood |
| Hematocrit | Ht | ~0.5 |  |
| Redistribution | k_α_ | 1.95x10^-4^/s | Fit for PS |
| Clearance | k_β_ | 6.55x10^-6^/s | Fit for PS |
| Fraction alpha | A | 57% | Fit for PS |
| Signal loss | k_loss_ | 1.28x10^-5^/s | 15 hr half life |
| Activation increase |  | 12 | Increase in fluorescence intensity upon cleavage |

*Antibody Uptake Model*

For antibody uptake simulations, the model has been described previously[18]. Due to the spatial heterogeneity of antibody uptake, a Krogh cylinder model was used where the uptake is a function of distance from the capillary source and time after injection. The equations that describe the concentration of unbound antibody, bound antibody-antigen complex, and free antigen are:

There is a no-flux or mirror boundary condition at the outer radius, since antibody diffusing away from one capillary diffuses towards another:

The boundary condition at the capillary surface depends on the permeability of the vessel[3] and diffusion into the tissue:

Using the method of lines, simulations were carried out using a stiff solver in Matlab (Mathworks; Natick, MA). The total antibody and bound concentration was integrated over the cylinder, yielding the average tumor concentration and fraction of antigen bound. The Krogh radius (R_Krogh_) was set to yield the overall blood vessel surface area to tumor volume ratio:

Table S3 – Parameter values for antibody simulations:

| **Symbol** | **Parameter** | **Figure 1** | **Figure 4** | **Note** |
| --- | --- | --- | --- | --- |
| [Ab]_p,0_ | Dose | 100 nM | - | 2 mL plasma volume |
| [Ag] | Antigen density | 1.7 μM | varied | 5x10^8^ cell/mL |
| P | Permeability | 3x10^-9^ m/s | - |  |
| S/V | Surface area to volume ratio | 100/cm | - | 20-200/cm |
| k_e_ | Internalization and loss rate | 1.3x10^-5^/s | - | Estimated [19] |
| A |  | 0.7 | - | Mouse IgG in mouse |
| k_alpha_ |  | 8x10^-6^/s | - |  |
| k_beta_ |  | 1.1x10^-6^/s | - |  |
| k_kappa_ | Normal capillary and lymphatic exchange | 3.9x10^-5^/s | - |  |
| k_lambda_ |  | 8.9x10^-5^/s | - |  |
| ε | Void fraction | 0.2 | - | Little effect on uptake |
| D | Diffusion coefficient | 14 μm^2^ | - |  |
| k_on_ | Association rate | 10^5^/Ms | - |  |
| k_off_ | Dissociation rate | 10^-4^/s | - |  |

(-) denotes same value for both

The resulting graph shows two regimes: a saturated regime on the left, and a subsaturating regime on the right. The transition point between these two regimes and the concentration in the subsaturating regime depend on multiple factors, such as the dose, permeability, and vascularization of the tumor. When both the clearance modulus and Thiele modulus[18] are less than one, the tumor will be in the saturation regime. Experimentally, this is often shown when tumors with very low expression levels of antigen have lower uptake than those with much higher expression. A large dose of ‘cold’ or non-fluorescent antibody can also block some of the sites, effectively lowering the antigen concentration and thereby lowering uptake of the labeled antibody.

**References**

1. Vaupel P, Kallinowski F, Okunieff P (1989) Blood-flow, oxygen and nutrient supply, and metabolic microenvironment of human-tumors - a review. Cancer Research 49: 6449-6465.

2. Yuan F, Dellian M, Fukumura D, Leunig M, Berk D, et al. (1995) Vascular Permeability in a Human Tumor Xenograft: Molecular Size Dependence and Cutoff Size. Cancer Research 55: 3752-3756.

3. Dreher MR, Liu WG, Michelich CR, Dewhirst MW, Yuan F, et al. (2006) Tumor vascular permeability, accumulation, and penetration of macromolecular drug carriers. Journal of the National Cancer Institute 98: 335-344.

4. Thurber GM, Schmidt MM, Wittrup KD (2008) Antibody tumor penetration: Transport opposed by systemic and antigen-mediated clearance. Advanced Drug Delivery Reviews 60: 1421-1434.

5. Besterman JM, Airhart JA, Low RB, Rannels DE (1983) Pinocytosis and intracellular degradation of exogneous protein - modulation by amino-acids. Journal of Cell Biology 96: 1586-1591.

6. Besterman JM, Airhart JA, Woodworth RC, Low RB (1981) Exocytosis of pinocytosed fluid in cultured-cells - kinetic evidence for rapid turnover and compartmentation. Journal of Cell Biology 91: 716-727.

7. Burgert HG, Thilo L (1983) Internalization and recycling of plasma-membrane glycoconjugates during pinocytosis in the macrophage cell-line, P388D1 - kinetic evidence for compartmentation of internalized membranes. Experimental Cell Research 144: 127-142.

8. Duncan R, Pratten MK, Cable HC, Ringsdorf H, Lloyd JB (1981) Effects of molecular-size of I-125-labeled poly(vinylpyrrolidone) on its pinocytosis by rat visceral yolk sacs and rat peritoneal-macrophages. Biochemical Journal 196: 49-55.

9. Walter RJ, Berlin RD, Pfeiffer JR, Oliver JM (1980) Polarization of endocytosis and receptor topography on cultured macrophages. Journal of Cell Biology 86: 199-211.

10. Almeida PC, Oliveira V, Chagas JR, Meldal M, Juliano MA, et al. (2000) Hydrolysis by cathepsin B of fluorescent peptides derived from human prorenin. Hypertension 35: 1278-1283.

11. Bogdanov AA, Lin CP, Simonova M, Matuszewski L, Weissleder R (2002) Cellular activation of the self-quenched fluorescent reporter probe in tumor microenvironment. Neoplasia 4: 228-236.

12. Griffiths G, Back R, Marsh M (1989) A quantitative-analysis of the endocytic pathway in baby hamster-kidney cells. Journal of Cell Biology 109: 2703-2720.

13. Mager DE (2006) Target-mediated drug disposition and dynamics. Biochemical Pharmacology 72: 1-10.

14. Baxter L, Zhu H, Mackensen D, Jain RK (1994) Physiologically Based Pharmacokinetic Model for Specific and Nonspecific Monoclonal Antibodies and Fragments in Normal Tissues and Human Tumor Xenografts in Nude Mice. Cancer Research 54: 1517-1528.

15. Nahrendorf M, Waterman P, Thurber G, Groves K, Rajopadhye M, et al. (2009) Hybrid In Vivo FMT-CT Imaging of Protease Activity in Atherosclerosis With Customized Nanosensors. Arteriosclerosis, Thrombosis, and Vascular Biology.

16. Davies PF, Ross R (1978) Mediation of Pinocytosis in Cultured Arterial Smooth-Muscle and Endothelial Cells by Platelet-Derived Growth-Factor. Journal of Cell Biology 79: 663-671.

17. Marsh M, Helenius A (1980) Adsorptive Endocytosis of Semliki Forest Virus. Journal of Molecular Biology 142: 439-454.

18. Thurber GM, Zajic SC, Wittrup KD (2007) Theoretic criteria for antibody penetration into solid tumors and micrometastases. J Nucl Med 48: 995-999.

19. Woo DV, Li DR, Mattis JA, Steplewski Z (1989) Selective chromosomal damage and cyto-toxicity of I-125-labeled monoclonal-antibody 17-1A in human cancer-cells. Cancer Research 49: 2952-2958.
